# Why excluding regenerative grazing skews beef’s carbon profile

**DOI:** 10.1073/pnas.2506941122

**Published:** 2025-05-30

**Authors:** Nataliya Apanovich, Deseret Weeks

**Affiliations:** ^a^School of Landscape Architecture and Planning, University of Arizona, Tucson, AZ 85716; ^b^Department of Sociology, Anthropology, and Criminal Justice, Utah State University, Logan, UT 84322

Dear Jonas Jagermeyr,

Eshel et al.’s (1) decision to exclude grazing on high carbon-sequestration lands rests on the assumption that all such land is better used for crops—but this is not universally supported by on-ground data. Not all carbon-rich grasslands are readily convertible to cropland, and some are already dedicated pasture where integrating rotational grazing can build soil carbon without displacing food production. In fact, while managed grazing of beef cattle may increase carbon sequestration potential of grasslands significantly, rotational grazing on marginal or degraded lands can substantially increase soil organic carbon, approaching levels seen in ungrazed ecosystems (2, 3). By omitting these positive-carbon scenarios, Eshel et al. (1) likely overstate grass-fed beef’s net emissions. Even the authors themselves concede that the most carbon-friendly grazing systems—those on fertile lands—could nearly neutralize beef’s emissions. Dismissing such cases as “misleading” overlooks a key insight: With appropriate land management or strategic use of certain lands, grass-fed beef production can sequester more soil organic carbon than continuous grazing and, as a component of regenerative agriculture, can contribute to climate change mitigation and a variety of ecosystem services production (4–6).

In short, while typical US rangeland beef is indeed emissions-intensive, the blanket assertion that all grass-fed beef is irrevocably high-carbon fails to account for the significant variability in outcomes driven by land choice and management.
